# Supplementary material for: A protocol for a pilot randomised controlled trial of an Early Psychiatric Assessment, Referral, and Intervention Study (EPARIS) for intensive care patients
Source: PLoS One. 2023 Jun 29;18(6):e0287470. doi: 10.1371/journal.pone.0287470 (PMC10309621; doi:10.1371/journal.pone.0287470)
Supplement: S1 Checklist — (DOCX) [file pone.0287470.s001.docx]

# Administrative Information

| Title {1} | 🗹 |
| --- | --- |
| Trial registration {2a and 2b}. | 🗹 |
| Protocol version {3} | 🗹 |
| Funding {4} | 🗹 |
| Author details {5a} | 🗹 |
| Name and contact information for the trial sponsor {5b} | 🗹 |
| Role of sponsor {5c} | 🗹 |

# Introduction

| Background and Rationale {6a, 6b} | 🗹 |
| --- | --- |
| Objectives {7} | 🗹 |
| Trial Design {8} | 🗹 |

# Methods

| Study Setting {9} | 🗹 |
| --- | --- |
| Eligibility Criteria {10} | 🗹 |
| Interventions {11a, 11b, 11c, 11d} | 🗹 |
| Outcomes {12} | 🗹 |
| Participant Timeline {13} | 🗹 |
| Sample Size {14} | 🗹 |
| Recruitment {15} | 🗹 |
| Allocation {16a, 16b, 16c} | 🗹 |
| Blinding {17a, 17b} | 🗹 |
| Data Collection {18a, 18b} | 🗹 |
| Data Management {19} | 🗹 |
| Statistical Methods {20a, 20b, 20c} | 🗹 |
| Data Monitoring {21a, 21b} | 🗹 |
| Harms {22} | 🗹 |
| Auditing {23} | 🗹 |
|  |  |
|  |  |
|  |  |
|  |  |

# Ethics and Dissemination

| Ethics Approval {24} | 🗹 |
| --- | --- |
| Protocol Amendments {25} | 🗹 |
| Consent or Assent {26a, 26b} | 🗹 |
| Confidentiality {27} | 🗹 |
| Declaration of Interests {28} | 🗹 |
| Access to Data {29} | 🗹 |
| Ancillary and Post Trial Care {30} | 🗹 |
| Dissemination Policy {31a, 31b} | 🗹 |

# Appendices

| Informed Consent and Materials {32} | 🗹 |
| --- | --- |
| Biological Specimens {33} | N/A |
