# Supplementary material for: A protocol for a pilot randomised controlled trial of an Early Psychiatric Assessment, Referral, and Intervention Study (EPARIS) for intensive care patients
Source: PLoS One. 2023 Jun 29;18(6):e0287470. doi: 10.1371/journal.pone.0287470 (PMC10309621; doi:10.1371/journal.pone.0287470)
Supplement: S1 Appendix — (DOC) [file pone.0287470.s002.doc]

# Study ID ______

# Metro North Patient Experience Survey – Outpatient and Community

|  | **Didn’t Apply** | **Never** | **Rarely** | **Sometimes** | **Mostly** | **Always** |
| --- | --- | --- | --- | --- | --- | --- |
| My views and concerns were listened to | ○ | ○ | ○ | ○ | ○ | ○ |
| My individual needs were met | ○ | ○ | ○ | ○ | ○ | ○ |
| When a need could not be met, staff explained why | ○ | ○ | ○ | ○ | ○ | ○ |
| I felt cared for | ○ | ○ | ○ | ○ | ○ | ○ |
| I was involved as much as I wanted in making decisions about my treatment and care | ○ | ○ | ○ | ○ | ○ | ○ |
| I was kept informed as much as I wanted about my treatment and care | ○ | ○ | ○ | ○ | ○ | ○ |
| As far as I could tell, the staff involved in my care communicated with each other about my | ○ | ○ | ○ | ○ | ○ | ○ |
| During waiting periods, before or after seeing my healthcare worker or team, I was able to occupy my time with enjoyable or useful activities | ○ | ○ | ○ | ○ | ○ | ○ |
|  |  | **Very Poor** | **Poor** | **Average** | **Good** | **Very Good** |
| Overall, the quality of the treatment and care I received was |  | ○ | ○ | ○ | ○ | ○ |

# LEC-5 Standard

Weathers, F., et al., *The life events checklist for DSM-5 (LEC-5).* 2013

**Instructions:** Listed below are a number of difficult or stressful things that sometimes happen to people. For each event check one or more of the boxes to the right to indicate that: (a) it happened to you personally; (b) you witnessed it happen to someone else; (c) you learned about it happening to a close family member or close friend; (d) you were exposed to it as part of your job (for example, paramedic, police, military, or other first responder); (e) you’re not sure if it fits; or (f) it doesn’t apply to you. Be sure to consider your entire life (growing up as well as adulthood) as you go through the list of events.

| **Event** | **Happened to me** | **Witnessed it** | **Learned about it** | **Part of my job** | **Not sure** | **Doesn’t apply** |
| --- | --- | --- | --- | --- | --- | --- |
| 1. Natural disaster (for example, flood, hurricane, tornado, earthquake) | ○ | ○ | ○ | ○ | ○ | ○ |
| 2. Fire or explosion | ○ | ○ | ○ | ○ | ○ | ○ |
| 3. Transportation accident (for example, car accident, boat accident, train wreck, plane crash) | ○ | ○ | ○ | ○ | ○ | ○ |
| 4. Serious accident at work, home, or during recreational activity | ○ | ○ | ○ | ○ | ○ | ○ |
| 5. Exposure to toxic substance (for example, dangerous chemicals, radiation) | ○ | ○ | ○ | ○ | ○ | ○ |
| 6. Physical assault (for example, being attacked, hit, slapped, kicked, beaten up) | ○ | ○ | ○ | ○ | ○ | ○ |
| 7. Assault with a weapon (for example, being shot, stabbed, threatened with a knife, gun, bomb) | ○ | ○ | ○ | ○ | ○ | ○ |
| 8. Sexual assault (rape, attempted rape, made to perform any type of sexual act through force or threat of harm) | ○ | ○ | ○ | ○ | ○ | ○ |
| 9. Other unwanted or uncomfortable sexual experience | ○ | ○ | ○ | ○ | ○ | ○ |
| 10. Combat or exposure to a war-zone (in the military or as a civilian) | ○ | ○ | ○ | ○ | ○ | ○ |
| 11. Captivity (for example, being kidnapped, abducted, held hostage, prisoner of war) | ○ | ○ | ○ | ○ | ○ | ○ |
| 12. Life-threatening illness or injury | ○ | ○ | ○ | ○ | ○ | ○ |
| 13. Severe human suffering | ○ | ○ | ○ | ○ | ○ | ○ |
| 14. Sudden violent death (for example, homicide, suicide) | ○ | ○ | ○ | ○ | ○ | ○ |
| 15. Sudden accidental death | ○ | ○ | ○ | ○ | ○ | ○ |
| 16. Serious injury, harm, or death you caused to someone else | ○ | ○ | ○ | ○ | ○ | ○ |
| 17. Any other very stressful event or experience | ○ | ○ | ○ | ○ | ○ | ○ |

# Posttraumatic Adjustment Screen

This questionnaire asks you questions that relate to factors that occurred before, during or after the event that caused your injuries. Circle the response that best describes how much you agree with the following statements.

O'donnell ML, Creamer MC, Parslow R, Elliott P, Holmes AC, Ellen S, Judson R, McFarlane AC, Silove D, Bryant RA. A predictive screening index for posttraumatic stress disorder and depression following traumatic injury. Journal of consulting and clinical psychology. 2008 Dec;76(6):923.

|  | **Not at all** | **To a small extent** | **To a moderate extent** | **To a large extent** | **Totally** |
| --- | --- | --- | --- | --- | --- |
| I have needed professional help to deal with emotional problems in the past. | ○ | ○ | ○ | ○ | ○ |
| Previously traumatic events have impacted negatively on my life in the past (e.g., assault, sexual abuse, previous combat duty, natural disasters, witnessing traumatic events). | ○ | ○ | ○ | ○ | ○ |
| In the past I was able to talk about my thoughts and feelings with my family members or friends. | ○ | ○ | ○ | ○ | ○ |
| In the past I was satisfied with the support that I had from my friends and family. | ○ | ○ | ○ | ○ | ○ |
| At the time of the event, I felt terrified, helpless or horrified. | ○ | ○ | ○ | ○ | ○ |
| During the event, I thought I was about to die. | ○ | ○ | ○ | ○ | ○ |
| I have felt irritable or angry since the event. | ○ | ○ | ○ | ○ | ○ |
| I have found it difficult to concentrate on what I was doing or things going on around me since the event. | ○ | ○ | ○ | ○ | ○ |
| I am confident that I can deal with the financial stressors that may arise as a consequence of being injured. | ○ | ○ | ○ | ○ | ○ |
| I can accept what happened to me | ○ | ○ | ○ | ○ | ○ |

# General Self Efficacy Scale

The General Self-Efficacy Scale is a 10-item psychometric scale that is designed to assess optimistic self-beliefs to cope with a variety of difficult demands in life.

Schwarzer, R., & Jerusalem, M. (1995). Generalized Self-Efficacy scale. In J. Weinman, S. Wright, & M. Johnston, Measures in health psychology: A user’s portfolio. Causal and control beliefs (pp. 35-37). Windsor, UK: NFER-NELSON

|  | **Not at all true** | **Hardly True** | **Moderately True** | **Exactly True** |
| --- | --- | --- | --- | --- |
| I can always manage to solve difficult problems if I try hard enough. | ○ | ○ | ○ | ○ |
| If someone opposes me, I can find the means and ways to get what I want. | ○ | ○ | ○ | ○ |
| It is easy for me to stick to my aims and accomplish my goals | ○ | ○ | ○ | ○ |
| I am confident that I could deal efficiently with unexpected events | ○ | ○ | ○ | ○ |
| Thanks to my resourcefulness, I know how to handle unforeseen situations | ○ | ○ | ○ | ○ |
| I can solve most problems if I invest the necessary effort | ○ | ○ | ○ | ○ |
| I can remain calm when facing difficulties because I can rely on my coping abilities | ○ | ○ | ○ | ○ |
| When I am confronted with a problem, I can usually find several solutions. | ○ | ○ | ○ | ○ |
| If I am in trouble, I can usually think of a solution. | ○ | ○ | ○ | ○ |
| I can usually handle whatever comes my way. | ○ | ○ | ○ | ○ |

# PCL-5

| Below is a list of problems that people sometimes have in response to a very stressful experience. Please read each problem carefully and then circle one of the numbers to the right to indicate how much you have been bothered by that problem in the past month. | | | | | |
| --- | --- | --- | --- | --- | --- |
| **In the past month, how much were you bothered by:** | **Not at all** | **A little bit** | **Moderately** | **Quite a bit** | **Extremely** |
| Repeated, disturbing, and unwanted memories of the stressful experience? | 0 | 1 | 2 | 3 | 4 |
| Repeated, disturbing dreams of the stressful experience? | 0 | 1 | 2 | 3 | 4 |
| Suddenly feeling or acting as if the stressful experience were actually happening again (as if you were actually back there reliving it)? | 0 | 1 | 2 | 3 | 4 |
| Feeling very upset when something reminded you of the stressful experience? | 0 | 1 | 2 | 3 | 4 |
| Having strong physical reactions when something reminded you of the stressful experience (for example, heart pounding, trouble breathing, sweating)? | 0 | 1 | 2 | 3 | 4 |
| Avoiding memories, thoughts, or feelings related to the stressful experience? | 0 | 1 | 2 | 3 | 4 |
| Avoiding external reminders of the stressful experience (for example, people, places, conversations, activities, objects, or situations)? | 0 | 1 | 2 | 3 | 4 |
| Trouble remembering important parts of the stressful experience? | 0 | 1 | 2 | 3 | 4 |
| Having strong negative beliefs about yourself, other people, or the world (for example, having thoughts such as: I am bad, there is something seriously wrong with me, no one can be trusted, the world is completely dangerous)? | 0 | 1 | 2 | 3 | 4 |
| Blaming yourself or someone else for the stressful experience or what happened after it? | 0 | 1 | 2 | 3 | 4 |
| Having strong negative feelings such as fear, horror, anger, guilt, or shame? | 0 | 1 | 2 | 3 | 4 |
| Loss of interest in activities that you used to enjoy? | 0 | 1 | 2 | 3 | 4 |
| Feeling distant or cut off from other people? | 0 | 1 | 2 | 3 | 4 |
| Trouble experiencing positive feelings (for example, being unable to feel happiness or have loving feelings for people close to you)? | 0 | 1 | 2 | 3 | 4 |
| Irritable behavior, angry outbursts, or acting aggressively? | 0 | 1 | 2 | 3 | 4 |
| Taking too many risks or doing things that could cause you harm? | 0 | 1 | 2 | 3 | 4 |
| Being “superalert” or watchful or on guard? | 0 | 1 | 2 | 3 | 4 |
| Feeling jumpy or easily startled? | 0 | 1 | 2 | 3 | 4 |
| Having difficulty concentrating? | 0 | 1 | 2 | 3 | 4 |
| Trouble falling or staying asleep? | 0 | 1 | 2 | 3 | 4 |

# The next few questions relate to the impact your ICU admission has had on your employment.

1. **What is your CURRENT employment status?**

- Employed full time
- Employed part time Looking for work (Skip to question 6)
- Unable to work due to reasons related to my ICU admission (Skip to question 7)
- Retired (Skip to question 7)
- Unable to work for other reasons (Questionnaire complete. Thank you.)

1. **What is your approximate annual income (before tax)?**

- Under $25,000
- Between $25,000 and $49,999
- Between $50,000 and $74,999
- Between $75,000 and $99,999
- More than $100,000

1. **How many sick days have you had to take over the last 12 months due to reasons related to your ICU admission?**

| - None | - 21-25 |
| --- | --- |
| - 1-4 | - 26-30 |
| - 5-10 | - 31-35 |
| - 11-15 | - More than 35 |
| - 16-20 |  |

1. **Do you believe your ICU admission has limited your employment options?**

- Yes – I have had to retrain
- Yes – it is more difficult to find work in my field
- No, there has been no impact on employment options

1. **If you are working part time, would you like to work more hours?**

- Yes, and I believe my ICU admission has prevented me from working as much as I would like
- Yes, but this is due to factors other than my ICU admission (Questionnaire complete. Thank you).
- No (Questionnaire complete. Thank you)
- N/A (I work full time)

1. **Did you retire earlier than planned due to your ICU admission?**

- Yes, between 1 to 5 years earlier than planned
- Yes, between 6 to 10 years earlier than planned
- Yes, more than 10 years earlier than planned
- No, I did not retire early due to my ICU admission.
- N/A (I am not retired)

# Post Appointment Questionnaire

AFTER your appointment, please indicate which statements best describe your experience today by placing a checkmark in one box in each group below.

|  | **Not at all** | **Slightly** | **Moderately** | **Quite** | **Extremely** |
| --- | --- | --- | --- | --- | --- |
| Overall, how helpful was your appointment today? | ○ | ○ | ○ | ○ | ○ |
| How helpful was your appointment in understanding any mental health symptoms you have been experiencing since leaving ICU or that may develop in future? | ○ | ○ | ○ | ○ | ○ |
| How confident are you about how to manage any mental health symptoms now or in future? | ○ | ○ | ○ | ○ | ○ |
| How confident are you about how to seek further support/treatment for your mental health now or in future? | ○ | ○ | ○ | ○ | ○ |
| How optimistic do you feel about your recovery? | ○ | ○ | ○ | ○ | ○ |
| How likely is it that you would recommend an appointment like this to a friend who has been in ICU? | ○ | ○ | ○ | ○ | ○ |
| Other comments/suggestions |  | | | | |

**Follow-up in 6 Months**

We would like to catch up with you in 6 months to see how you are progressing. This will involve completing some of the same questionnaires as you have completed today.

If you are happy to be contacted in 6 months, please provide the best ways to contact you below:

- Email _________________________
- Phone _________________________
- Postal Address _________________________

_________________________

_________________________
