# Supplementary material for: A protocol for a pilot randomised controlled trial of an Early Psychiatric Assessment, Referral, and Intervention Study (EPARIS) for intensive care patients
Source: PLoS One. 2023 Jun 29;18(6):e0287470. doi: 10.1371/journal.pone.0287470 (PMC10309621; doi:10.1371/journal.pone.0287470)
Supplement: S2 Appendix — (DOC) [file pone.0287470.s003.doc]

# Health Services Use

| **Over the past month how many times have you seen a:** | | | | | | | | | | | | **Did you find this helpful with your mental health?** |
| --- | --- | --- | --- | --- | --- | --- | --- | --- | --- | --- | --- | --- |
|  | **0** | **1** | **2** | **3** | **4** | **5** | **6** | **7** | **8** | **9** | **10+** |  |
| General Practitioner | ○ | ○ | ○ | ○ | ○ | ○ | ○ | ○ | ○ | ○ | ○ | ○ Yes ○ No |
| Psychiatrist | ○ | ○ | ○ | ○ | ○ | ○ | ○ | ○ | ○ | ○ | ○ | ○ Yes ○ No |
| Psychologist | ○ | ○ | ○ | ○ | ○ | ○ | ○ | ○ | ○ | ○ | ○ | ○ Yes ○ No |
| Medical Specialist | ○ | ○ | ○ | ○ | ○ | ○ | ○ | ○ | ○ | ○ | ○ | ○ Yes ○ No |
| Social Worker | ○ | ○ | ○ | ○ | ○ | ○ | ○ | ○ | ○ | ○ | ○ | ○ Yes ○ No |
| Counsellor (eg. Marriage counsellor) | ○ | ○ | ○ | ○ | ○ | ○ | ○ | ○ | ○ | ○ | ○ | ○ Yes ○ No |
| District or community nurse | ○ | ○ | ○ | ○ | ○ | ○ | ○ | ○ | ○ | ○ | ○ | ○ Yes ○ No |
| Chiropractor | ○ | ○ | ○ | ○ | ○ | ○ | ○ | ○ | ○ | ○ | ○ | ○ Yes ○ No |
| Physiotherapist | ○ | ○ | ○ | ○ | ○ | ○ | ○ | ○ | ○ | ○ | ○ | ○ Yes ○ No |
| Alternative therapist (eg. Acupuncturist, herbalist, naturopath, masseur, homeopath) | ○ | ○ | ○ | ○ | ○ | ○ | ○ | ○ | ○ | ○ | ○ | ○ Yes ○ No |
| Other (please specify) | ○ | ○ | ○ | ○ | ○ | ○ | ○ | ○ | ○ | ○ | ○ | ○ Yes ○ No |
| If you think you needed help/more help from health professionals for your mental health and didn't get it, why didn't you get this help? Please choose the main reason or the single reason that most closely applies. | - I preferred to manage myself - I didn't think anything (more) could help - I didn't know where to get (more) help - I was afraid to ask for help, or of what others would think of me if I did - I couldn't afford the money - I couldn’t physically get to the appointments - I asked, but didn't get the help - I got help from another source, please specify:   _________________________________________   - Other, please specify   _________________________________________ | | | | | | | | | | | |

# General Self Efficacy Scale

The General Self-Efficacy Scale is a 10-item psychometric scale that is designed to assess optimistic self-beliefs to cope with a variety of difficult demands in life.

Schwarzer, R., & Jerusalem, M. (1995). Generalized Self-Efficacy scale. In J. Weinman, S. Wright, & M. Johnston, Measures in health psychology: A user’s portfolio. Causal and control beliefs (pp. 35-37). Windsor, UK: NFER-NELSON

|  | **Not at all true** | **Hardly True** | **Moderately True** | **Exactly True** |
| --- | --- | --- | --- | --- |
| I can always manage to solve difficult problems if I try hard enough. | ○ | ○ | ○ | ○ |
| If someone opposes me, I can find the means and ways to get what I want. | ○ | ○ | ○ | ○ |
| It is easy for me to stick to my aims and accomplish my goals | ○ | ○ | ○ | ○ |
| I am confident that I could deal efficiently with unexpected events | ○ | ○ | ○ | ○ |
| Thanks to my resourcefulness, I know how to handle unforeseen situations | ○ | ○ | ○ | ○ |
| I can solve most problems if I invest the necessary effort | ○ | ○ | ○ | ○ |
| I can remain calm when facing difficulties because I can rely on my coping abilities | ○ | ○ | ○ | ○ |
| When I am confronted with a problem, I can usually find several solutions. | ○ | ○ | ○ | ○ |
| If I am in trouble, I can usually think of a solution. | ○ | ○ | ○ | ○ |
| I can usually handle whatever comes my way. | ○ | ○ | ○ | ○ |

# EQ-5D

By placing a checkmark in one box in each group below, please indicate which statements best describe your own health state today.

**Mobility**

- I have no problems in walking about
- I have some problems in walking about
- I am confined to bed

**Self-Care**

- I have no problems with self-care
- I have some problems washing or dressing myself
- I am unable to wash or dress myself

**Usual Activities (e.g. work, study, housework, family or leisure activities)**

- I have no problems with performing my usual activities
- I have some problems with performing my usual activities
- I am unable to perform my usual activities

**Pain/Discomfort**

- I have no pain or discomfort
- I have moderate pain or discomfort
- I have extreme pain or discomfort

**Anxiety/Depression**

- I am not anxious or depressed
- I am moderately anxious or depressed
- I am extremely anxious or depressed

Hospital Anxiety and Depression Scale (HADS)

**Instructions:** Doctors are aware that emotions play an important part in most illnesses. If your doctor knows about these feelings, he or she will be able to help you more. This questionnaire is designed to help your doctor know how you feel.

Read each item and circle the reply which comes closest to how you have been feeling in the past week. Don’t take too long over your replies: your immediate reaction to each item will probably be more accurate than a long thought-out response.

| **I feel tense or ‘wound up’:** | **A** |  | **I feel as if I am slowed down:** | **D** |
| --- | --- | --- | --- | --- |
| Most of the time | 3 |  | Nearly all of the time | 3 |
| A lot of the time | 2 |  | Very often | 2 |
| Time to time, occasionally | 1 |  | Sometimes | 1 |
| Not at all | 0 |  | Not at all | 0 |
| **I still enjoy the things I used to enjoy:** | **D** |  | **I get a sort of frightened feeling like ‘butterflies in the stomach’:** | **A** |
| Definitely as much | 0 |  | Not at all | 0 |
| Not quite so much | 1 |  | Occasionally | 1 |
| Only a little | 2 |  | Quite often | 2 |
| Not at all | 3 |  | Very often | 3 |
| **I get a sort of frightened feeling like something awful is about to happen:** | **A** |  | **I have lost interest in my appearance:** | **D** |
| Very definitely and quite badly | 3 |  | Definitely | 3 |
| Yes, but not too badly | 2 |  | I don’t take as much care as I should | 2 |
| A little, but it doesn’t worry me | 1 |  | I may not take quite as much care | 1 |
| Not at all | 0 |  | I take just as much care as ever | 0 |
| **I can laugh and see the funny side of things:** | **D** |  | **I feel restless as if I have to be on the move:** | **A** |
| As much as I always could | 0 |  | Very much indeed | 3 |
| Not quite so much now | 1 |  | Quite a lot | 2 |
| Definitely not so much now | 2 |  | Not very much | 1 |
| Not at all | 3 |  | Not at all | 0 |
| **Worrying thoughts go through my mind:** | **A** |  | **I look forward with enjoyment to things:** | **D** |
| A great deal of the time | 3 |  | A much as I ever did | 0 |
| A lot of the time | 2 |  | Rather less than I used to | 1 |
| From time to time but not too often | 1 |  | Definitely less than I used to | 3 |
| Only occasionally | 0 |  | Hardly at all | 2 |
| **I feel cheerful:** | **D** |  | **I get sudden feelings of panic:** | **A** |
| Not at all | 3 |  | Very often indeed | 3 |
| Not often | 2 |  | Quite often | 2 |
| Sometimes | 1 |  | Not very often | 1 |
| Most of the time | 0 |  | Not at all | 0 |
| **I can sit at ease and feel relaxed:** | **A** |  | **I can enjoy a good book or radio or TV programme:** | **D** |
| Definitely | 0 |  | Often | 0 |
| Usually | 1 |  | Sometimes | 1 |
| Not often | 2 |  | Not often | 2 |
| Not at all | 3 |  | Very seldom | 3 |

# PCL-5

| Below is a list of problems that people sometimes have in response to a very stressful experience. Please read each problem carefully and then circle one of the numbers to the right to indicate how much you have been bothered by that problem in the past month. | | | | | |
| --- | --- | --- | --- | --- | --- |
| **In the past month, how much were you bothered by:** | **Not at all** | **A little bit** | **Moderately** | **Quite a bit** | **Extremely** |
| Repeated, disturbing, and unwanted memories of the stressful experience? | 0 | 1 | 2 | 3 | 4 |
| Repeated, disturbing dreams of the stressful experience? | 0 | 1 | 2 | 3 | 4 |
| Suddenly feeling or acting as if the stressful experience were actually happening again (as if you were actually back there reliving it)? | 0 | 1 | 2 | 3 | 4 |
| Feeling very upset when something reminded you of the stressful experience? | 0 | 1 | 2 | 3 | 4 |
| Having strong physical reactions when something reminded you of the stressful experience (for example, heart pounding, trouble breathing, sweating)? | 0 | 1 | 2 | 3 | 4 |
| Avoiding memories, thoughts, or feelings related to the stressful experience? | 0 | 1 | 2 | 3 | 4 |
| Avoiding external reminders of the stressful experience (for example, people, places, conversations, activities, objects, or situations)? | 0 | 1 | 2 | 3 | 4 |
| Trouble remembering important parts of the stressful experience? | 0 | 1 | 2 | 3 | 4 |
| Having strong negative beliefs about yourself, other people, or the world (for example, having thoughts such as: I am bad, there is something seriously wrong with me, no one can be trusted, the world is completely dangerous)? | 0 | 1 | 2 | 3 | 4 |
| Blaming yourself or someone else for the stressful experience or what happened after it? | 0 | 1 | 2 | 3 | 4 |
| Having strong negative feelings such as fear, horror, anger, guilt, or shame? | 0 | 1 | 2 | 3 | 4 |
| Loss of interest in activities that you used to enjoy? | 0 | 1 | 2 | 3 | 4 |
| Feeling distant or cut off from other people? | 0 | 1 | 2 | 3 | 4 |
| Trouble experiencing positive feelings (for example, being unable to feel happiness or have loving feelings for people close to you)? | 0 | 1 | 2 | 3 | 4 |
| Irritable behavior, angry outbursts, or acting aggressively? | 0 | 1 | 2 | 3 | 4 |
| Taking too many risks or doing things that could cause you harm? | 0 | 1 | 2 | 3 | 4 |
| Being “superalert” or watchful or on guard? | 0 | 1 | 2 | 3 | 4 |
| Feeling jumpy or easily startled? | 0 | 1 | 2 | 3 | 4 |
| Having difficulty concentrating? | 0 | 1 | 2 | 3 | 4 |
| Trouble falling or staying asleep? | 0 | 1 | 2 | 3 | 4 |

#

# The next few questions relate to the impact your ICU admission has had on your employment.

1. **What is your CURRENT employment status?**

- Employed full time
- Employed part time Looking for work (Skip to question 6)
- Unable to work due to reasons related to my ICU admission (Skip to question 7)
- Retired (Skip to question 7)
- Unable to work for other reasons (Questionnaire complete. Thank you.)

1. **What is your approximate annual income (before tax)?**

- Under $25,000
- Between $25,000 and $49,999
- Between $50,000 and $74,999
- Between $75,000 and $99,999
- More than $100,000

1. **How many sick days have you had to take over the last 12 months due to reasons related to your ICU admission?**

| - None | - 21-25 |
| --- | --- |
| - 1-4 | - 26-30 |
| - 5-10 | - 31-35 |
| - 11-15 | - More than 35 |
| - 16-20 |  |

1. **Do you believe your ICU admission has limited your employment options?**

- Yes – I have had to retrain
- Yes – it is more difficult to find work in my field
- No, there has been no impact on employment options

1. **If you are working part time, would you like to work more hours?**

- Yes, and I believe my ICU admission has prevented me from working as much as I would like
- Yes, but this is due to factors other than my ICU admission (Questionnaire complete. Thank you).
- No (Questionnaire complete. Thank you)
- N/A (I work full time)

1. **Did you retire earlier than planned due to your ICU admission?**

- Yes, between 1 to 5 years earlier than planned
- Yes, between 6 to 10 years earlier than planned
- Yes, more than 10 years earlier than planned
- No, I did not retire early due to my ICU admission.
- N/A (I am not retired)

# 
