# Supplementary material for: A protocol for a pilot randomised controlled trial of an Early Psychiatric Assessment, Referral, and Intervention Study (EPARIS) for intensive care patients
Source: PLoS One. 2023 Jun 29;18(6):e0287470. doi: 10.1371/journal.pone.0287470 (PMC10309621; doi:10.1371/journal.pone.0287470)
Supplement: S1 File — (DOCX) [file pone.0287470.s004.docx]

Research Protocol

Department name

Service line

Location line 1
Location line 2

Contact phone (optional)

Contact email (optional)

Facility/hospital/clinical service name

**Early Psychiatric Assessment and Referral Intervention Study (E-PARIS)**

**Investigators and responsibilities**

| **Name** | **Role** | **Department** |
| --- | --- | --- |
| A/ Prof. Dylan Flaws | Psychiatrist  Head of Mental Health Research  Associate Professor | Metro North Mental Health  Critical Care Research Group  Faculty of Clinical Sciences, QUT |
| Dr. Stuart Baker | Intensivist | Redcliffe Intensive Care |
| Charlene Wall | ICU Nurse | Redcliffe Intensive Care |
| A/Prof. Alexis Tabah | Intensivist | Redcliffe Intensive Care |
| Dr. Hamish Pollock | Director of Intensive Care | Redcliffe Intensive Care |
| Dr. Mahesh Ramanan | Intensivist | Caboolture Intensive Care |
| Kylie Jacobs | ICU Nurse Researcher  Psychologist | Redcliffe Intensive Care |
| Dr. Tracey Varker | Senior Research Fellow | Department of Psychiatry, University of Melbourne |
| Dr. Olivia Metcalf | Research Fellow | Department of Psychiatry, University of Melbourne |
| Prof. Adrian Barnett | Statistician | QUT |
| A/Prof. Susan Patterson | Research Fellow | Critical Care Research Group |

# Resources

This contributes to a Metro North Clinician Research Fellowship awarded to A/Prof Dylan Flaws to improve recovery outcomes after an admission to the intensive care unit (ICU). He is able to provide his input to the intervention as an in-kind contribution.

The study will be conducted in the Redcliffe Post Intensive Care Clinic, with representatives from Intensive Care, Psychiatry, and Allied Health in the investigative team. The study will be operating on the in-kind contributions from the investigators, and existing infrastructure already established in the clinic. The study team brings the complementary expertise and authority needed to deliver the study.

# Background and rationale

Clinical research and technological innovation have greatly improved survival after ICU admission such that around 90% of those admitted to ICUs are discharged alive [1]. As a result of this, there has been a shift in outcome focus from survival to quality of life after survival. Evidence demonstrates that recovery is often complicated and partial [2], with up to 80% of people discharged from the ICU (ICU survivors) experiencing various physical, cognitive, and/or psychological complications and enduring impairment which can persist up to 15 years after discharge [3-5]. These various impairments, occurring separately or in combination following an ICU admission are termed ‘post intensive care syndrome’ (PICS).

A recent concept analysis of PICS defined its attributes as new or worsening multidimensional impairments, physical dysfunction, psychological disorder, cognitive impairment, failed social reconstruction or impaired multidimensional symptoms which persist beyond ICU and hospital discharge[6].

Mental illness is a common feature of PICS. A large cohort study of 4,943 ICU survivors in the UK found the prevalence of anxiety, depression, and PTSD to be 46%, 40% and 22% respectively, with 18% meeting criteria for all three conditions [7]. A recent study also found an increased prevalence of mental illness treatment and psychotropic medication use in the 5 years following an ICU admission when compared to the 5 years prior [8].

The prevalence and burden of PICS remains under described in Australia [9], and may vary between ICU populations, but available data demonstrate that it is associated with substantial personal and ﬁnancial burden for patients and families[10]. Disrupting social engagement and employment, health and social costs are immense [3, 11, 12]. Specifically, an association has been found between post-ICU psychological morbidity and poor quality of life (QoL) across all domains regardless of aetiology[13].

With the current evidence base for PICS being scarce internationally, and available evidence demonstrating that current interventions are not effective longer-term; further research into prevention, early diagnosis and intervention of this syndrome is considered internationally as a priority [14]. Many post-ICU clinics review patients sometime after discharge, but to our knowledge the effect of an early psychiatric intervention soon after ICU discharge on PICS outcomes has not been investigated. This is despite a recent meta-analysis demonstrating benefit from early psychological intervention targeted to symptomatic individuals following recent trauma[15] and a recent overview of PICS emphasising the importance of early screening and treatment of psychological morbidity to prevent longer term psychological impairment, and suggesting that future research in post-ICU clinics needs to find ways for a more targeted approach to intervention delivery[16].

We hypothesise that an early psychiatric intervention involving evaluation, initial treatment (such as psychoeducation and pharmacotherapy), and referral is feasible and practicable to implement in a post-ICU clinic and acceptable to that patient population Such an intervention may contribute to an improved sense of self-efficacy, improved access of ongoing therapy, and a subsequent reduction in the long-term burden of PICS – both for the individual patients needing this care, and on broader health systems.

# Aims and objectives

The primary objective of this pilot RCT study is to:

1. To evaluate feasibility (cost and practicability) and acceptability of an early psychiatric intervention in a post-ICU clinic.

Secondary Objectives are:

1. Assess the impact of early psychiatric assessment and provision of referral options on self-efficacy and rates of health service access for patients requiring psychological intervention post-ICU
2. Measure the change in symptom severity for anxiety, depression, and PTSD between presentation, and 6 months after presentation.
3. Measure the impact on work, income, and independence in activities of daily living (ADL)

# Setting

This study will be conducted in the Redcliffe Post Intensive Care Clinic. Redcliffe Hospital is a busy metropolitan hospital in Brisbane, Queensland. The Redcliffe Hospital ICU has 10 beds and admits 500 patients per year. It provides general intensive care management for a broad spectrum of critical illnesses, apart from tertiary cardiothoracic, neurosurgical, trauma and burns management.

Redcliffe Hospital has an established ICU follow-up clinic, where ICU survivors are reviewed around three months after discharge from hospital and extensive data is collected on outcomes from critical illness. Patients are referred to the post-ICU clinic predominantly from the Redcliffe Hospital ICU, but also at times from other local hospital ICUs.

The clinic operates every Wednesday from 0800 to 1600 and reviews around 80 patients per year. The clinician is responsible for making initial contact with patients recently discharged from Intensive Care who meet engagement criteria (ICU admission over 48 hours and no cognitive impairment that prevents participation).  This occurs by phone within the first 1 to 3 months from discharge as a brief encounter to engage with the service. The administrative officer prepares a list of patients to be contacted.

The Redcliffe post-ICU clinic provides an ideal opportunity for this pilot RCT, as it is one of very few post-ICU clinics currently operating in Australia, with processes for data collection and follow-up embedded into standard practice. There has also been growing interest in post-ICU clinics in Australia, with new clinics being planned in several states. As such, the findings of this study provide an opportunity to inform post-ICU care both locally and nationally.

Diverse research and clinical eﬀorts to improve the experience and outcomes of patients admitted to ICUs are ongoing within Metro North HHS. This study is complementary to those efforts and extends collaboration between the units, other directorates (mental health), across disciplines, and academic partners.

# Study plan / participants and recruitment

**Study design:**

This pilot study is a prospective, single site, parallel randomised controlled trial. Data will be collected with consent from routinely kept hospital records, and from participants at two times as specified below.

**Participants and sample calculation:**

Data will be collected from a sample of approximately 30 patients attending the Redcliffe ICU follow-up clinic over a 12-month period, with 15 randomised to the control and intervention arms respectively.

**PICS Feature Measure Minimally Clinically Important Difference (MCID)**

Anxiety/Depression HADS* ≥ 2.5 points[17]

PTSD PCL-5 ≥ 10 points[18]

Physical Function EQ5D* ≥ 0.5 points[19]

*Collected as part of routine clinic practices

**Inclusion criteria:**

People discharged from the participating ICU will be eligible to participate if they are:

- Meets the Post-ICU Clinic criteria
  - ICU admission >48hrs
  - Nil cognitive impairment that prevents participation
- Aged 18 years or over
- Residing in Australia
- Sufficiently fluent in English to complete recruitment and data collection processes
- Able to provide a GP with which the research team can correspond about the intervention
- Likely to be contactable at the 6-month follow-up time

**Exclusion criteria:**

- Patients whose life expectancy at discharge is estimated by their clinical team to be less than 6 months
- Unable or unwilling to provide consent to participate for the duration of the study
- Provide consent, but do not provide baseline or 6-month follow-up data
- Unlikely to remain contactable for 6 months after their clinic appointment

## Sampling and recruitment

Patients attending the Redcliffe Post-ICU clinic will be informed about the trial of an inclusion of an early psychiatric review as part of the clinic following their completion of standard clinic cares (see Appendix 1 for the Standard Operating Procedures).

If the patient expresses interest in the study, they will be provided written and verbal information regarding the purpose and aims of the study and invited to ask any questions they have about the study before being invited to participate. Potential participants will not be aware if they have been randomised to the control or intervention arm at this stage.

The potential participant will be provided multiple opportunities to discuss the study with members of staff not involved in the study. Information will be provided in writing and verbally before the patient is invited to consent to participate in the study.

A patient information and consent form will be provided. People who agree to participate will complete the written consent form before any data collection takes place. Patients who consent to participate will be randomised using a simple randomisation schedule. Participants randomised to the intervention arm will be offered an appointment with the psychiatrist in addition to standard clinic cares, whereas participants randomised to the control arm will receive standard clinic cares only.

For those attending their appointment over the phone, verbal consent will be obtained and witnessed. People who give consent will give permission for research staff to access data from their clinical records and will complete a series of questionnaires examining their premorbid function, experiences in ICU and their subsequent recovery across a range of domains.

To monitor recruitment, a re-identifiable log will be kept that will document patients eligible for approach, and subsequent contact.

Participants will be able to withdraw from the study at any time. If a participant that has been randomised to receive the early psychiatric intervention chooses to withdraw consent, they will receive the standard cares provided by the clinic without the intervention, and any collected data will be withdrawn from the study.

Six months after clinic presentation, research staff will contact all participants to offer a follow-up appointment either in the clinic or by phone and conduct the follow-up survey as described below. Participants will be invited to continue and will have the opportunity to withdraw from the study before this additional data is collected.

At the 6-month follow-up timepoint when potential participants do not respond to the first contact attempt, research staff will attempt contact again one week later. Potential participants who remain uncontactable after the second attempt will be considered lost to follow-up and not contacted again.

# Study plan

## ICU Information Bundle

Relevant information routinely collected during the patient’s ICU admission will be accessed via the ICU clinic information system.

## Initial Survey

Participants will first be provided a series of questionnaires after their standard clinic appointment as described below. The initial survey can be found in Appendix 2.

### Background information

The following questionnaires have been selected to provide information on the patient’s premorbid risk/protective factors and baseline level of function.

The “Metro North Patient Experience Survey – outpatient and community” is an 11-question patient reported experience measure (**PREM**) and will be used to collect information about the participant’s experience during their ICU admission.

The Life Events Checklist (**LEC**) for DSM-5 is a self-report measure designed to screen for potentially traumatic events in a respondent's lifetime. The LEC-5 assesses exposure to 16 events known to potentially result in PTSD or distress and includes one additional item assessing any other extraordinarily stressful event not captured in the first 16 items[20]. There is no formal scoring protocol or interpretation per se, other than identifying whether a person has experienced one or more of the events listed. Respondents indicate varying levels of exposure to each type of potentially traumatic event included on a 6-point nominal scale, and respondents may endorse multiple levels of exposure to the same trauma type. The LEC-5 does not yield a total score or composite score.

The Posttraumatic Adjustment Screen (**PAS**) is a 10-item screening index that has been designed to facilitate the early identification of individuals most at risk for developing PTSD and/or depression following a traumatic injury.

The **General Self Efficacy Scale** is a 10-item psychometric scale that is designed to assess optimistic self-beliefs to cope with a variety of difficult demands in life[21].

The **Patient Employment Information Questionnaire** has been amended from a Questionnaire previously used in epilepsy research and reworded to relate to the patient’s ICU admission. It consists of seven questions around changes to employment and income since their ICU admission.

### PICS Measures

Participants will be invited to complete a suite of carefully selected measures encompassing physical, and psychological function, henceforth referred to as the PICS Measures. As indicated below, some of these measures are collected as part of BAU.

The following research tools have been selected as they have been found to be valid and reliable in this patient cohort and the majority recommended for use in measuring outcomes after ICU admission by an expert group after a DELPHI survey [22]. The research team have beta tested this battery of questions with a control sample, demonstrating that completion takes 13-20 minutes.

The **Hospital Anxiety and Depression Scale (HADS)*** is a questionnaire consisting of two subscales measuring patients’ symptoms of anxiety and depression. Each subscale consists of seven items scored from 0 to 3, resulting in a subscale score range from 0 to 21. A subscale score above 7 suggests clinically significant problems. The questionnaire has been validated among general medical patients as well as critically ill patients [23].

The PTSD Checklist for DSM-5 (**PCL-5**) is a 20-item self-report measure that assesses the 20 DSM-5 symptoms of PTSD. The PCL-5 is a self-report measure that can be completed by patients in a waiting room prior to a session or by participants as part of a research study. It takes approximately 5-10 minutes to complete[24].

The **EQ-5D-5L*** is a standardised, non-disease specific instrument for measuring health-related quality of life and is validated in a critical care population. It consists of a 5-question questionnaire. The five domains assessed are mobility, self-care, usual activities, pain/discomfort, and anxiety/depression[25].

*Collected as part of routine clinic practices and thus not included Appendix 2.

## Post Appointment Questionnaire

To address the primary outcome, the **Post Appointment Questionnaire** is an 8 item self-reported acceptability measure that has been specifically developed for this study. It has been adapted from similar surveys used in previous acceptability studies. Questions focus on whether the participant found their appointment helpful, how confident they feel about managing any psychological symptoms they have or may develop, and how optimistic they feel about their recovery.

## Follow-up Survey

At the 6-month timepoint, participants will repeat the Post Appointment Questionnaire, PICS Measures (including HADS and EQ5D-5L), Life Events Checklist, Generalised Self Efficacy Screen and Patient Employment Information Questionnaire, as well as the Health Services Use Questionnaire described below. The follow-up Survey can be found in Appendix 3.

This **Health Services Use** Questionnaire has 11-items and been adapted from a questionnaire utilised in a recent injury vulnerability study[26]. It explores how often the participant has attended a range of clinical, allied health and alternative health services, whether these services were helpful for their mental health, and for those think they needed more help from health professionals than was accessed, the main reason they didn’t get this help.

# Study procedure

Following verbal consent being provided, participants will be randomised to attend the clinic either when the psychiatrist is present or absent block randomisation in blocks of 4 or 6, in a 1:1 ratio, with the list generated by the study statistician (AB) and provided in enclosed envelopes to conceal allocation until verbal consent is provided. Full written consent will then be obtained when they attend their appointment.

Data will be collected with participant permission from their clinical records and medical history through a data request to ICU data collectors and hospital coders. These data will include demographics, frailty scores, diagnoses and severity of illness, previous mental health history, medications, multidisciplinary interventions, evidence of delirium during their admission and in-hospital complications.

| **Time points** | **Data collected** | |
| --- | --- | --- |
| Discharge from ICU | Contacted by clinical staff about the clinic. | |
| Clinic Appointment | Consent to participate, PICF completed. Routine Hospital Data accessed. Initial survey provided. Randomised to intervention or control arm. | |
|  | Control Group  Standard clinic cares provided as described below. | Intervention Group  Early psychiatric intervention provided as described below, in addition to standard clinic cares. |
| Intervention Follow-up |  | Participant contacted to evaluate response/tolerability of intervention. GP provided further advice as detailed below. |
| 6 Month Follow-up | Participant provides consent to continue participating and completes Follow-up Survey. | |

The timeframe of data collection is summarised in the table below:

## Standard Clinic Cares

As per the Standard Operating Procedure, patients attending the clinic participate in an interview, screening and assessment process followed by the opportunity to provide feedback. This is described in brief below. A comprehensive description can be found in Appendix 1.

**Conducting an Interview**

1. ICU admission reviewed to understand context, severity of illness and possible ailments.
2. Patient contacted through face to face, telehealth or telephone appointment.
3. Introduction and purpose of interview

Example: *“Thank you for coming in, the clinic runs to provide additional support to patients discharged from ICU. Research is being conducted to help us understand the impact our treatment is having on those surviving ICU admissions.”*

1. Gain confirmation to discuss admission details and conduct assessments.

Example: *“Do you recall your admission in ICU? Would you like me to explain what occurred?”*

If a patient declines and does not wish to talk about admission details, this is respected and staff move onto the assessment phase of the process.

1. Timeline of admission; from origin of admission to discharge. Key points and treatments received (i.e. Intubation, CPR, Medications received, OT, Extubation) are discussed.
2. Assessment form and standardised tools completed (see below):.

Routine Assessments

EQ-5D-5L

HADS

IES-R

Katz index of independence in activities of daily living

Montreal Cognitive Assessment / MOCA – BLIND

**Assessment**

History

- Electronic medical record and discharge summary reviewed.
- Events surrounding initial presentation used to begin the conversation about admission.
- ICU events such as issues and interventions encountered discussed.
- Staff are respectful of a patient's decision not to revisit their hospital admission in detail.

Assessment

- Assessment of body systems
- Patient asked to self-assess their quality of life and functional ability pre and post ICU
- Further assessment and referral to specialist services considered as appropriate.

Psycho- social

- Social history encompassing pre and post ICU admission
- Questions of relationships, employment, housing, and social interactions. Staff review services that may be able to alleviate additional stress.
- Patient's recollection of their admission explored, mindful that some patients will not remember anything about their time in ICU while others recall vivid memories.
- Acknowledging their feelings and experiences by explaining the prevalence and causes of hallucinations and delusions
- Staff consider possible referral for counselling services.

Feedback

- Review current processes.
- Explore services that may be required.

## Early Psychiatric Assessment and Referral Intervention

**General Assessment**

Participants randomised to the intervention group will attend the clinic on a day and time when the psychiatrist is in attendance. Consenting patients will see the psychiatrist after completing the standard clinical cares described above.

Prior to seeing the participant, the psychiatrist will review the clinical notes pertaining to their premorbid history, and their ICU admission. In addition, the psychiatrist will review the results of the baseline questionnaires, ICU notes and other relevant clinical information. The psychiatrist will discuss these results with the participant and explore how this information interrelates with their premorbid history, and their ICU experience.

The psychiatrist will conduct a comprehensive psychiatric review, including comorbid disorders, substance use, suicidal ideation, psychosocial stressors, social/emotional supports.

Diagnoses of any mental illnesses present will be made based on DSM-V criteria. Psychoeducation will be provided on any new mental illness present, with detail on how this interrelates with their critical illness recovery, and general lifestyle advice will be provided (exercise, healthy eating, sleep hygiene).

Initial treatment for any new mental illness commonly seen in post-ICU populations will be provided as described below.

**Management of Anxiety Disorder**

Anxiety disorders will be managed according to the Royal Australian and New Zealand College of Psychiatrists guidelines[27]. A brief synopsis is provided below.

Mild Severity – Education provided for Cognitive Behavioural Therapy (CBT), and letter sent to GP to make referral.

Moderate Severity – Referral for CBT as above or Medication* or CBT plus Medication

Severe – CBT plus medication

*As per RANZCP Guidelines Selective Serotonin Reuptake Inhibitor (SSRI) or Serotonin and Noradrenaline Reuptake Inhibitor (SNRI) antidepressants will be offered as first line in consultation with patient’s preferences and comorbidities. Where these cannot be utilised, Mirtazapine will also be considered.

**Management of Mood Disorder**

As with Anxiety disorders, Mood disorders will be managed according to the respective Royal Australian and New Zealand College of Psychiatrists guidelines for Mood Disorders[28]. A brief synopsis is provided below.

Psychological Treatments

Education will be provided on approved psychological treatments for mood disorders:

CBT, Interpersonal Therapy (IPT), Problem-Solving Therapy, Behavioral Activation Therapy, Nondirective Supportive Therapy, and Short-Term Psychodynamic Psychotherapy.

Pharmacological Treatments

Pharmacological treatment will be considered in context with the patient’s medical comorbidities, preferences and prominent mood symptoms. RANZCP guidelines are summarised in the table below.

| **Prominent Symptom(s)** | **Preferred Medication** |
| --- | --- |
| Anxiety | SSRI/SNRI |
| Cognitive Difficulties | Duloxetine, Vortioxetine |
| Sleep Disturbance | Mirtazapine, Agomelatine |
| Fatigue | Bupropion |
| Pain | Duloxetine, Tricyclic Antidepressant (TCA) |
| Melancholia | TCA |

**Management of Post-Traumatic Stress Disorder (PTSD)**

As with Anxiety disorders and Mood disorders, PTSD will be managed according to the respective Royal Australian and New Zealand College of Psychiatrists guidelines for PTSD, utilising a stepped care approach. A brief synopsis is provided below.

Psychological Treatments

Where indicated, education will be provided on Trauma Focused CBT (TF-CBT), and Eye Movement Desensitisation and Reprogramming (EMDR). Letter sent to GP to make referral for the agreed-on therapy.

Pharmacological Treatments

Pharmacological treatment will be discussed in circumstances where any of the following applies:

- The participant is unwilling or not in a position to engage in or access recommended psychological therapy.
- The participant has a comorbid condition or associated symptoms (e.g., clinically significant depression and high levels of dissociation) where SSRIs are indicated.
- The participant’s circumstances are not sufficiently stable to commence recommended psychological therapy (as a result, for example, of significant ongoing life stress such as domestic violence).
- The participant has not gained significant benefit from recommended psychological therapy.
- There is a significant wait time before psychological treatment is available.

First line medications considered will be SSRIs and SNRIs. Additional medications with evidence for PTSD such as Prazosin and Quetiapine will be considered where appropriate.

**Mental Illness not related to ICU presentation**

Where a patient has a pre-existing mental illness which is already being managed by their GP or other service, no further intervention will be provided.

Where a new mental illness is identified or suspected, but unrelated to their ICU admission, contact details for relevant services will be provided to the participant and the psychiatrist will notify the patient’s GP of the diagnosis and advise referral for ongoing review and treatment as indicated.

**Intervention Follow-up**

The Psychiatrist will follow-up all patients where an intervention is initiated 4-6 weeks after their appointment. If there has been least partial clinical response, a letter will be sent to the participant’s GP recommending continuing current cares with ongoing monitoring. If there has been no response or a non-urgent deterioration, a letter will be sent to the GP suggesting referral for ongoing psychiatric review.

**Imminent risks**

If at clinic assessment the participant appears to require inpatient psychiatric treatment, or there is concern of an imminent risk such as (suicidal ideation with intent and plan), or an urgent clinical deterioration, the participant will be directed to the Redcliffe Emergency Department for immediate review by their local mental health service.

If the imminent risk is identified during the intervention follow-up, the participant will be encouraged to attend their local hospital for urgent review by the mental health service.

## 6 Month Outcomes Follow-up

At the completion of data collection at the clinic appointment, permission will be sought to contact participants again 6 months after their appointment and participants will be asked to nominate a preferred form of contact.

A member of the research team will contact the participant via their preferred contact method approximately 6 months after their clinic appointment.

After providing consent to continue participating, the participant will complete the outcome measure bundle again.

## Data management and security:

All data will be treated in confidence and only made accessible to members of the research team on an as needs basis. All paper records will be stored in a locked filing cabinet, and any electronic databases will be stored on password protected computers. For dissemination of results, participants will only be referred to in the coded form e.g. “Participant A”, “Participant B” etc. All records will be destroyed (permanently deleted or shredded as appropriate) after 15 years, as per ‘Good Clinical Practice’ (GCP) guidelines.

Electronically collected data will be directly entered into REDCap, hosted on a Queensland Health server. Hard copy responses will be manually entered into REDCap by a member of the research team. Once recruitment is complete, Data will be cleaned and uploaded to statistical software package R for analysis.

## Statistical analysis

The demographics and results of the Baseline Outcome Measures Bundle will be described using proportions and associated 95% confidence intervals and compared between intervention and control groups to check the integrity of the randomisation.

Responses to the Post Appointment Questionnaire will be assumed to be additive and averaged to produce the primary outcome measure. Differences between groups will be assessed using t-test.

Changes in secondary outcome measures between baseline and 6 months will be compared between intervention and control groups to identify any clinically and statistically significant differences using ANCOVA regression with patients results at baseline fitted as a covariate. The model residuals will be checked to look for outliers and bi-modality. Leave-one-out sensitivity analyses will be used to look for patients that strongly influence the results.

Two-by-two tables will be created of the predicted and observed PICS, together with statistics on sensitivity, specificity, and negative and positive predictive value. Models will be checked for influential observations and outliers. The results will be reported using the EQUATOR TRIPOD checklist to ensure completeness and transparency[29].

The overall characteristics of the sample will be described using summary statistics. This will help inform the generalisability of our results. The number of patients approached and consented will be tabulated, together with the number who dropped out or died.

.

# Ethical Considerations

Ethical issues relevant to the study and procedures to promote best practice are outlined below.

All participants will be made aware that any participation is completely voluntary.

**Consent:**

All potential participants will receive information about the study in writing and verbally and have the opportunity to ask any questions they have and discuss their participation with a person of their choosing should they wish. Potential Participants will be allowed as much time as they need to consider participation, and participants can decline assessments or withdraw from further participation at any time.

The research team decided for simple randomisation prior to consent for the following reasons:

Minimise departure from standard practice for participants not participating in the study.

In BAU, patients are not invited to the post-ICU clinic while still in ICU, and this is instead discussed several weeks after discharge to allow some time for rest and recovery prior. Informed consent while still in hospital would require participants to make a decision about the post-ICU clinic much earlier in their recovery trajectory than usual.

Respect the patient’s right to distributive justice

The research team were concerned that because ICU clinical staff cannot be blinded to when the intervention is available, a non-structured approach to appointment allocation risks unconscious bias from staff. While a departure from BAU, simple randomisation would ensure that patients’ rights to equal opportunity in care and to participate in research is respected.

Respect the patient’s right to sufficient opportunity to consider participation prior to consent

In BAU, participants are invited to attend the post-ICU clinic via telephone several weeks after discharge. The research team felt that invitation to participate, or even to randomisation in this setting would not provide the patient sufficient time to reflect and seek advice before making a decision.

**Confidentiality and privacy:**

First approach to patients regarding the study will be by a member of the treating team who will seek permission to introduce a researcher.

Data will be de-identified at source and stored using research code numbers. Information linking named participants to these code numbers will be kept separately from any research databases and archived for fifteen years once data collection has been completed.

**Participant safety:**

The study will be conducted in accord with the principles of the Helsinki Declaration and International, Australian and Queensland legislation and Queensland Health policies and procedures. Full ethical approval will be sought prior to collection or analysis of research specific data. Ethical approval will be sought from the RBWH Human Research Ethics Committee, and Research Governance approval will subsequently be applied for.

However, all participants will be provided with contact details for services where they can seek support if required. If we identify an acute deterioration in mental state or imminent risk, the participant will be directed to attend the local hospital for review by the relevant mental health service, as described in the study procedure.

We are aware of the potential for some people who agree to contact at either time point to have become unwell or die before the next contact. In all cases, follow-up contact will be handled sensitively.

# Dissemination and outcome

Results will be published in peer reviewed publications and presented at professional conferences. Results will also be presented to local decision makers and in appropriate local consumer forums.

Participants will be asked if they wish to receive plain English summaries of the results from the study. Plain English summaries will be posted to interested participants.

As there is a paucity of evidence in this area, the results of this study will inform future studies seeking to estimate the burden of disease of PICS in Metro North HHS and continue to develop a targeted intervention for PICS applicable to both Metro North hospitals, nationally and internationally.

# References

1. Zimmerman, J.E., A.A. Kramer, and W.A. Knaus, *Changes in hospital mortality for United States intensive care unit admissions from 1988 to 2012.* Critical care, 2013. **17**(2): p. R81.

2. Needham, D.M., et al., *Improving long-term outcomes after discharge from intensive care unit: report from a stakeholders' conference.* Critical care medicine, 2012. **40**(2): p. 502-509.

3. Davidson, J.E. and M.A. Harvey, *Patient and Family Post-Intensive Care Syndrome.* AACN Adv Crit Care, 2016. **27**(2): p. 184-6.

4. Wang, S., et al., *Aging and Post-Intensive Care Syndrome: A Critical Need for Geriatric Psychiatry.* Am J Geriatr Psychiatry, 2018. **26**(2): p. 212-221.

5. Pandharipande, P.P., et al., *Long-term cognitive impairment after critical illness.* New England Journal of Medicine, 2013. **369**(14): p. 1306-1316.

6. Yuan, C., F. Timmins, and D.R. Thompson, *Post-intensive care syndrome: A concept analysis.* International Journal of Nursing Studies, 2021. **114**: p. 103814.

7. Hatch, R., et al., *Anxiety, depression and post traumatic stress disorder after critical illness: a UK-wide prospective cohort study.* Critical care, 2018. **22**(1): p. 1-13.

8. Olafson, K., et al., *The 5-year pre-and post-hospitalization treated prevalence of mental disorders and psychotropic medication use in critically ill patients: a Canadian population-based study.* Intensive Care Medicine, 2021. **47**(12): p. 1450-1461.

9. Rawal, G., S. Yadav, and R. Kumar, *Post-intensive Care Syndrome: an Overview.* J Transl Int Med, 2017. **5**(2): p. 90-92.

10. Kang, J., Y.J. Jeong, and J. Hong, *The effect of postintensive care syndrome on the quality of life of intensive care unit survivors: A secondary analysis.* Australian Critical Care, 2021. **34**(3): p. 246-253.

11. Griffiths, J., et al., *An exploration of social and economic outcome and associated health-related quality of life after critical illness in general intensive care unit survivors: a 12-month follow-up study.* Crit Care, 2013. **17**(3): p. R100.

12. Swoboda, S.M. and P.A. Lipsett, *Impact of a prolonged surgical critical illness on patients' families.* Am J Crit Care, 2002. **11**(5): p. 459-66.

13. da Costa, J.B., et al., *Psychological disorders in post-ICU survivors and impairment in quality of life.* Psychology & Neuroscience, 2019. **12**(3): p. 391.

14. Rosa, R.G., et al., *Effects of post-ICU follow-up on subject outcomes: A systematic review and meta-analysis.* Journal of critical care, 2019.

15. Roberts, N.P., et al., *Early psychological intervention following recent trauma: A systematic review and meta-analysis.* European journal of psychotraumatology, 2019. **10**(1): p. 1695486.

16. Ramnarain, D., et al., *Post Intensive Care Syndrome (PICS): An overview of the definition, etiology, risk factors, and possible counseling and treatment strategies.* Expert review of neurotherapeutics, 2021. **21**(10): p. 1159-1177.

17. Chan, K.S., et al., *Distribution-based estimates of minimal important difference for hospital anxiety and depression scale and impact of event scale-revised in survivors of acute respiratory failure.* General hospital psychiatry, 2016. **42**: p. 32-35.

18. PTSD Checklist for DSM-5 (PCL-5). 2017 [cited 2022 March 31st]; Available from: <https://www.ptsd.va.gov/professional/assessment/adult-sr/ptsd-checklist.asp>.

19. Coretti, S., M. Ruggeri, and P. McNamee, *The minimum clinically important difference for EQ-5D index: a critical review.* Expert review of pharmacoeconomics & outcomes research, 2014. **14**(2): p. 221-233.

20. Weathers, F., et al., *The life events checklist for DSM-5 (LEC-5).* 2013.

21. Schwarzer, R. and M. Jerusalem, *Generalized self-efficacy scale.* J. Weinman, S. Wright, & M. Johnston, Measures in health psychology: A user’s portfolio. Causal and control beliefs, 1995. **35**: p. 37.

22. Dinglas, V.D., L.N. Faraone, and D.M. Needham, *Understanding patient-important outcomes after critical illness: a synthesis of recent qualitative, empirical, and consensus-related studies.* Curr Opin Crit Care, 2018. **24**(5): p. 401-409.

23. Milton, A., et al., *Early psychological screening of intensive care unit survivors: a prospective cohort study.* Crit Care, 2017. **21**(1): p. 273.

24. Weathers, F.W., et al., *The ptsd checklist for dsm-5 (pcl-5).* Scale available from the National Center for PTSD at www. ptsd. va. gov, 2013. **10**(4).

25. Roll, M.A., et al., *Long-term survival and health-related quality of life in adults after extra corporeal membrane oxygenation.* Heart, Lung and Circulation, 2019. **28**(7): p. 1090-1098.

26. Bryant, B.M., et al., *Evaluating Patient-Level Medication Regimen Complexity Over Time in Heart Transplant Recipients.* Annals of Pharmacotherapy, 2016. **50**(11): p. 926-934.

27. Andrews, G., et al., *Royal Australian and New Zealand College of Psychiatrists clinical practice guidelines for the treatment of panic disorder, social anxiety disorder and generalised anxiety disorder.* Australian & New Zealand Journal of Psychiatry, 2018. **52**(12): p. 1109-1172.

28. Malhi, G.S., et al., *The 2020 Royal Australian and New Zealand College of Psychiatrists clinical practice guidelines for mood disorders.* Australian & New Zealand Journal of Psychiatry, 2021. **55**(1): p. 7-117.

29. <https://www.equator-network.org/reporting-guidelines/tripod-statement/>.
