# Supplementary material for: A protocol for a pilot randomised controlled trial of an Early Psychiatric Assessment, Referral, and Intervention Study (EPARIS) for intensive care patients
Source: PLoS One. 2023 Jun 29;18(6):e0287470. doi: 10.1371/journal.pone.0287470 (PMC10309621; doi:10.1371/journal.pone.0287470)
Supplement: Attachment — Submitted filename: Response to Reviewers 120123.docx [file pone.0287470.s005.docx]

PONE-D-22-25386

A Protocol for a Pilot Randomised Controlled Trial of an Early Psychiatric Assessment, Referral, and Intervention Study (EPARIS) for Intensive Care Patients

PLOS ONE

Dear Dr. Dylan Finlay Flaws,

Thank you for submitting your manuscript to PLOS ONE. After careful consideration, we feel that it has merit but does not fully meet PLOS ONE’s publication criteria as it currently stands. Therefore, we invite you to submit a revised version of the manuscript that addresses the points raised during the review process.

Please submit your revised manuscript by Feb 19 2023 11:59PM. If you will need more time than this to complete your revisions, please reply to this message or contact the journal office at [plosone@plos.org](mailto:plosone@plos.org). When you're ready to submit your revision, log on to <https://www.editorialmanager.com/pone/> and select the 'Submissions Needing Revision' folder to locate your manuscript file.

Please include the following items when submitting your revised manuscript:

- A rebuttal letter that responds to each point raised by the academic editor and reviewer(s). You should upload this letter as a separate file labeled 'Response to Reviewers'.
- A marked-up copy of your manuscript that highlights changes made to the original version. You should upload this as a separate file labeled 'Revised Manuscript with Track Changes'.
- An unmarked version of your revised paper without tracked changes. You should upload this as a separate file labeled 'Manuscript'.

If you would like to make changes to your financial disclosure, please include your updated statement in your cover letter. Guidelines for resubmitting your figure files are available below the reviewer comments at the end of this letter.

If applicable, we recommend that you deposit your laboratory protocols in protocols.io to enhance the reproducibility of your results. Protocols.io assigns your protocol its own identifier (DOI) so that it can be cited independently in the future. For instructions see: <https://journals.plos.org/plosone/s/submission-guidelines#loc-laboratory-protocols>. Additionally, PLOS ONE offers an option for publishing peer-reviewed Lab Protocol articles, which describe protocols hosted on protocols.io. Read more information on sharing protocols at <https://plos.org/protocols?utm_medium=editorial-email&utm_source=authorletters&utm_campaign=protocols>.

We look forward to receiving your revised manuscript.

Kind regards,

Alison Wang
Academic Editor
PLOS ONE

Journal Requirements:

When submitting your revision, we need you to address these additional requirements.

1. Please ensure that your manuscript meets PLOS ONE's style requirements, including those for file naming. The PLOS ONE style templates can be found at 
<https://journals.plos.org/plosone/s/file?id=wjVg/PLOSOne_formatting_sample_main_body.pdf> and 
<https://journals.plos.org/plosone/s/file?id=ba62/PLOSOne_formatting_sample_title_authors_affiliations.pdf>

*Thank you, we have consulted the formatting style and ensured the manuscript meets PLOS One’s requirements. Please let us know if you require anything to be changed.*

2. Please include captions for your Supporting Information files at the end of your manuscript, and update any in-text citations to match accordingly. Please see our Supporting Information guidelines for more information: <http://journals.plos.org/plosone/s/supporting-information>.

*We have added a list of supporting documents to the end of the manuscript and checked that the body of the manuscript refers to these accurately.*

3. We note that the original protocol that you have uploaded as a Supporting Information file contains an institutional logo. As this logo is likely copyrighted, we ask that you please remove it from this file and upload an updated version upon resubmission.

*All institutional logos have now been removed. We have also removed appendices that contain the surveys due to licensing issues. All measures from the surveys are still cited in Table 2 and referenced.*

Additional Editor Comments:
1. Please further detail the methods of this study, including the methods of randomization, concealment, blinding.

*A new section has been added to specifically describe the process of allocation and concealment. It has also been clarified that this is an open label RCT due to the nature of the intervention.*

2. please add one section to elaborate how to maintain the intervention fidelity of this study.

*We have added a section discussing the process of maintaining intervention fidelity, including how unforeseen situations will be handled and how this will be published with the study findings.*

[Note: HTML markup is below. Please do not edit.]

Reviewers' comments:

Reviewer's Responses to Questions

**Comments to the Author**

1. Does the manuscript provide a valid rationale for the proposed study, with clearly identified and justified research questions?

The research question outlined is expected to address a valid academic problem or topic and contribute to the base of knowledge in the field.

Reviewer #1: Yes

Reviewer #2: Yes

2. Is the protocol technically sound and planned in a manner that will lead to a meaningful outcome and allow testing the stated hypotheses?

The manuscript should describe the methods in sufficient detail to prevent undisclosed flexibility in the experimental procedure or analysis pipeline, including sufficient outcome-neutral conditions (e.g. necessary controls, absence of floor or ceiling effects) to test the proposed hypotheses and a statistical power analysis where applicable. As there may be aspects of the methodology and analysis which can only be refined once the work is undertaken, authors should outline potential assumptions and explicitly describe what aspects of the proposed analyses, if any, are exploratory.

Reviewer #1: Yes

Reviewer #2: Yes

3. Is the methodology feasible and described in sufficient detail to allow the work to be replicable?


Descriptions of methods and materials in the protocol should be reported in sufficient detail for another researcher to reproduce all experiments and analyses. The protocol should describe the appropriate controls, sample size calculations, and replication needed to ensure that the data are robust and reproducible.

Reviewer #1: Yes

Reviewer #2: Yes

4. Have the authors described where all data underlying the findings will be made available when the study is complete?

The [PLOS Data policy](https://journals.plos.org/plosone/s/materials-and-software-sharing) requires authors to make all data underlying the findings described in their manuscript fully available without restriction, with rare exception, at the time of publication. The data should be provided as part of the manuscript or its supporting information, or deposited to a public repository. For example, in addition to summary statistics, the data points behind means, medians and variance measures should be available. If there are restrictions on publicly sharing data—e.g. participant privacy or use of data from a third party—those must be specified.

Reviewer #1: No

Reviewer #2: No

*Data availability has now been clarified at the end of the Statistical Plan section.*

5. Is the manuscript presented in an intelligible fashion and written in standard English?

PLOS ONE does not copyedit accepted manuscripts, so the language in submitted articles must be clear, correct, and unambiguous. Any typographical or grammatical errors should be corrected at revision, so please note any specific errors here.

Reviewer #1: Yes

Reviewer #2: Yes

6. Review Comments to the Author

Please use the space provided to explain your answers to the questions above and, if applicable, provide comments about issues authors must address before this protocol can be accepted for publication. You may also include additional comments for the author, including concerns about research or publication ethics.

You may also provide optional suggestions and comments to authors that they might find helpful in planning their study.


(Please upload your review as an attachment if it exceeds 20,000 characters)

Reviewer #1: This is a protocol paper for a pilot trial. Keeping up with the goal of the pilot trial the paper describes what the feasibility and acceptability of the pilot RCT type paper should target. My suggestion to only change is that author must either remove the ANCOVA analysis description or must state what is the purpose of that analysis. As a pilot RCT this must not target for establishing or testing any statistical anything.

*We acknowledge the reviewer’s concerns, as while Statistical analysis can be used in pilot studies and be appropriate, problems can arise when people create p-values and start testing hypotheses. We have clarified both in the abstract, and in section 4.1 – Outcome Measures that we won’t test hypotheses in this study, but we still want to fit appropriate statistical models to get an idea of the size of the effect and best inform the effect size for the larger sample size calculation.*
The other revision I suggest is the writing style. For example, almost all sections consist of too many small paragraphs each consisting of 2-4 sentences. Also, provide section numbers for ease of readability and references.

*The manuscript has been revised to improve readability with better paragraphing and section numbers.*

Reviewer #2: This paper will contribute to a pragmatic evaluation of the acceptability of introducing early psychiatric assessment into an existing post-ICU follow-up process, and if considered acceptable, will inform future research into the efficacy and generalisability of the intervention. However, some changes are advised in order to improve the transparency for the reader:

1. -Was the sample size calculated upon previous studies? It remains unclear whether appropriate power calculations were made prior to recruiting patients. Please take this up in your methods section or in the discussion.

*This is a convenience sample based on the availability of the psychiatrist to administer the intervention for the purposes of this study for 12 months. This has now been clarified in Section 3.4 – Participants and Sample Size*

2. can you provide further details regarding how to recruit participants and research team members?

*This is now described in more detail in section 3.4 – Study Team, Participants and Sample Size*

3. Page 10: No cognitive impairment that prevents participation: please be concise, what scale are you talking about here? It would be good to name this scale and add the appropriate reference of this scale.

*This has been reworded as “and able to provide informed consent”*

4. What will be the primary outcomes and the secondary outcomes for this pilot RCT, and how to measure these outcomes? Please provide more details about the outcome measurements.

*The authors have intentionally avoided describing a “primary outcome measure” due to this being a pilot RCT. However, section 4.1 “Outcome Measure” has been reworded to try and clarify which outcome measures are being collected, and with which measurement tools. For further clarity, these have now been subdivided into “4.1.1 - Feasibility and acceptability outcomes”, and “4.1.2 – Treatment outcomes”.*

5. Please provide information about the roles of research team members. For example, who will be responsible for the intervention delivery? Who will be responsible for the data collection?

*This is now better described in section 3.4 – Study Team, Participants and Sample Size.*

6. as stated in Page 17:” Estimate the effect size of any treatment effects to inform power calculations of future studies evaluating whether the intervention is beneficial”, how to calculate the effect size of any treatment effects? Are you going to use cohen’s d or Hedges's g?

*The purpose of this pilot is to inform subsequent studies that will inform patient care. As such, our intention is to keep all measures focused on patient outcome. For this reason, the investagors have elected for MCID rather than Cohen’s d or Hedge’s g as a measure of effect size. Section 4.1.2 clarifies the rationale for this choice and refers the reader to Table 4 for the MCID of each outcome variable.*

7. as stated in Page 17 “The amount of wave and item-missing data will be tabulated by treatment group. We will impute item missing data using multiple imputation.” Please justify why multiple imputation will be used to deal with the missing data, instead of another commonly used method- last observation carried forward (LOCF).

*Section 4.3 The authors elected for multiple imputation as LOCF relies on a strong assumption of no change and makes no use of potentially valuable information about the participants and/or time. Simulation studies have shown that it does not perform as well as multiple imputation for dealing with missing data (see for example DOI: 10.1002/sim.2099 and DOI: 10.1080/10543400903242795 and https://pubmed.ncbi.nlm.nih.gov/15889392/).*

8. please provide the information of “Data management”. Who can access all the collected data? And where are you going to store and dispose of the research data hard copies?

*Section 4.4 – Data Management and Security now describes how physical and electronic data will be managed during the study, and how findings and deidentified data will be made available after study completion.*
