# Supplementary material for: A protocol for a pilot randomised controlled trial of an Early Psychiatric Assessment, Referral, and Intervention Study (EPARIS) for intensive care patients
Source: PLoS One. 2023 Jun 29;18(6):e0287470. doi: 10.1371/journal.pone.0287470 (PMC10309621; doi:10.1371/journal.pone.0287470)
Supplement: Attachment — Submitted filename: Response to Reviewers 040423.docx [file pone.0287470.s006.docx]

Reviewer #3: I am late to this party. I wonder if the chosen acceptability outcome will really reflect acceptability. The analysis will, of course, be carried on people who accepted to participate in the study. Among those who refused, some may have issues of acceptance of a psychiatric intervention. I guess the authors will have the opportunity to discuss this in their final paper. A qualitative approach might provide a deeper understanding of the acceptability and the overall experience of meeting a psychiatrist.

*We acknowledge the limitations of a quantitative approach evaluating the acceptability of the intervention, and this will be reflected in the way the findings are interpreted and reported. We have made a small amendment to the acceptability question to provide the participant to specifically comment on whether they would recommend adding a psychiatric review to current clinic cares. The freetext question at the bottom of the post-appointment questionnaire may provide some insights, but the authors feel a dedicated qualitative study is likely to be required following this study.*

Reviewer #4: Thank you for giving me the opportunity to review this protocol manuscript. The authors propose an interesting intervention that if feasible, could greatly impact the well-being of ICU patients.

I was however surprised that the authors went for a quantitative approach for this pilot study. With such a small expected sample size and given the objectives, focus groups or interviews with patients and healthcare providers might have been more pertinent. At the very least, I would recommend to carefully document any comments from patients, psychiatrist, and other healthcare provider to strengthen the evaluation of the feasibility and acceptability of the psychiatric intervention.

*As mentioned in response to reviewer 3, we acknowledge the limitations of a quantitative approach, and will interpret our findings accordingly. We will take note of free-text responses, but feel a dedicated qualitative study will need to follow the current study. This is now discussed in limitations.*

This being said, I appreciate how the authors remain humble and transparent regarding sample size, statistical power, and analyses. For instance, the sample size and participation rate estimation are very well described: “The feasible recruitment window is 12 months, with an estimated maximum possible sample size of 80. Assuming a 50% recruitment percentage, it is estimated that data will be collected from a sample of approximately 30 to 40 patients attending the Redcliffe ICU follow-up clinic over 12 months, with 15 to 20 allocated to the control and intervention arms respectively.” However, they do not mention any expectation regarding dropout between appointment and 6-months post-appointment time. I would advise to give an approximation of the expected dropout when describing the expected sample size.

*This has been clarified as being the estimate for the whole process. The manuscript now elaborates a 70% recruitment rate, and 70% retention rate.*

I would advise to also measure the LEC-5 at 6-months post appointment to ensure that no new adverse life event could explain a big change at follow-up.

*Thank you for this suggestion. The LEC-5 has now been added to the follow-up survey.*

The authors mention how and when the psychiatrist will refer to the patients’ GP, and all in all it seems that a lot is put on the hands of the GPs. Thus, the authors might want to also include the GPs’ perspective when evaluating feasibility and acceptability of the intervention. Also, the “Health Service Use” variable results at follow-up will depend quite substantially on the reactivity of the patient’s GP.

*This is a very important question, and one the authors have discussed thoroughly. We looked at how we could modify the current study to capture this information but are concerned that it would not produce data of a sufficient quality to answer the question. As such, we feel that a dedicated study will be required to explore the feasibility and acceptability of the intervention from the GP perspective, perhaps combined with the aforementioned qualitative study. We now discuss this in the limitations section.*

Could the authors please provide a reference for the MCID they plan to use? And ideally mention whether and how those thresholds have been validated.

*The MCID are tabulated in table 4, and cited to the following references:*

*Chan KS, Friedman LA, Bienvenu OJ, Dinglas VD, Cuthbertson BH, Porter R, et al. Distribution-based estimates of minimal important difference for hospital anxiety and depression scale and impact of event scale-revised in survivors of acute respiratory failure. General hospital psychiatry. 2016;42:32-5.*

*National Center for PTSD. ; 2017 [Available from:* [*https://www.ptsd.va.gov/professional/assessment/adult-sr/ptsd-checklist.asp*](https://www.ptsd.va.gov/professional/assessment/adult-sr/ptsd-checklist.asp)*.*

*Coretti S, Ruggeri M, McNamee P. The minimum clinically important difference for EQ-5D index: a critical review. Expert review of pharmacoeconomics & outcomes research. 2014;14(2):221-33.*

*If there are other MCIDs or additional references the reviewer would suggest, we would be happy to provide them.*

Other minor comments:
- I would advise the authors to avoid abbreviations in the Abstract or clearly state their meaning the first time they are mentioned in the abstract.

*All abbreviations have been removed or stated in the abstract.*

- Please provide the full name of the EQ5D-5L somewhere.

*We have never seen EQ-5D written in full, but have elaborated in section 4.1.2 that it refers to the EuroQOL survey (5 domains, 5 levels).*

- In Table 3, the term “Systems” is not clear.

*We have elaborated with some examples of body systems to assist the reader in understanding what is being reviewed in this part of the assessment.*

- “Participants will also give permission for research staff to access data from their clinical records and will complete a series of questionnaires as described below.” I think the authors mean “above”

*This has been amended.*
