# Supplementary material for: A protocol for a pilot randomised controlled trial of an Early Psychiatric Assessment, Referral, and Intervention Study (EPARIS) for intensive care patients
Source: PLoS One. 2023 Jun 29;18(6):e0287470. doi: 10.1371/journal.pone.0287470 (PMC10309621; doi:10.1371/journal.pone.0287470)
Supplement: Attachment — Submitted filename: Response to Reviewers 010623.docx [file pone.0287470.s007.docx]

To whom it may concern,

We hope the attached manuscript has addressed the comments and provided a more detailed justification for the MCIDs used in this study, as well as the rationale for a quantitative pilot study prior to a subsequent qualitative or nested study.

Reviewer #4: I thank the authors for their answer to my comments and the revisions undertaken. I found that they addressed satisfactorily my concerns, except for two minor points:
1. I now see the references for the MCIDs and I would still encourage the author to mention their validation in the text. For instance, by stating that the HADS’ MCID was validated in a survivors of acute respiratory failure sample using distribution-based methods and that it showed comparable estimates across studies, follow-up, and country.

*Thank you, we have now elaborated in section 4.1.2 how the MCID values listed in table 4 were validated.*

2. Mentioning a potential qualitative follow-up investigation is a good think. I would additionally indicate why a quantitative exploration is an important first step.

*We have elaborated in section 6 that a quantitative pilot has been selected in the first instance to inform subsequent larger studies, with subsequent or nested qualitative studies intended.*

Thank you for taking the time to review this manuscript, and please let us know if you have any further questions.

Warm Regards,

A/Prof. Dylan Flaws
